# P-1490. Evaluating the Impact of Inadequate Empiric Gram-Negative Antibiotic Therapy on Clinical Outcomes

**DOI:** 10.1093/ofid/ofae631.1660

**Published:** 2025-01-29

**Authors:** Jonathan Baghdadi, Katherine E Goodman, Laurence S Magder, Kimberly C Claeys, Mark Sutherland, Anthony Harris

**Affiliations:** University of Maryland School of Medicine, Baltimore, Maryland; University of Maryland School of Medicine, Baltimore, Maryland; University of Maryland, Baltimore, MD; University of Maryland Baltimore, Baltimore, Maryland; University of Maryland School of Medicine, Baltimore, Maryland; University of Maryland School of Medicine, Baltimore, Maryland

## Abstract

**Background:**

Many clinicians select broad-spectrum antibiotic therapy based on the concern that inadequate empiric coverage will be associated with a worse outcome. To explore this concern, we compared outcomes between patients started on empiric narrow-spectrum antibiotics and later escalated to broad-spectrum antibiotics v. patients started empirically on broad-spectrum antibiotics and then continued on broad-spectrum antibiotics.

**Figure 1.**
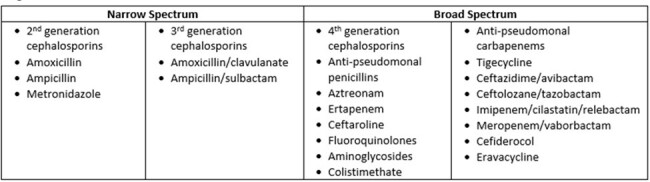
Antibiotic Classification

**Methods:**

We conducted a retrospective observational cohort study of adult patients discharged in 2019 from 120 hospitals in the Premier Healthcare Database. Encounters for adult patients were included if associated with (1) initiation of narrow- or broad-spectrum Gram-negative antibiotics on hospital day 0-2 and (2) administration of a broad-spectrum Gram-negative antibiotic as definitive therapy (see **Fig. 1** for classification of antibiotics as narrow v. broad). Patients who received empiric narrow-spectrum antibiotics were matched to patients who received empiric broad-spectrum antibiotics using propensity scores. A win ratio was estimated to compare a ranked composite outcome including mortality, readmission, and adverse drug events between the two groups (see **Fig. 2** for outcome structure).

**Figure 2.**
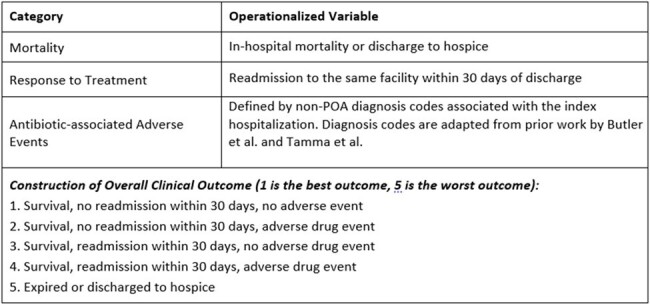
Components of Overall Clinical Outcome including Desirability of Outcome Ranking (DOOR)

**Results:**

81,826 patients who received empiric narrow-spectrum antibiotics were matched to 81,826 patients who received empiric broad-spectrum antibiotics (see **Table 1** for patient characteristics). Mortality was lower (8.9% v. 9.2%, p = 0.022), readmission was less frequent (11.1% v. 12.7%, p < 0.0001), and adverse events were more frequent (10.5% v. 9.0%, p < 0.0001) after empiric narrow-spectrum antibiotics than empiric broad-spectrum antibiotics (see **Table 2** for outcomes). Head-to-head comparison of ranked outcomes between the two groups suggested better clinical outcomes associated with empiric narrow-spectrum antibiotics (win ratio 1.04, p = 0.0007).

**Table 1.**
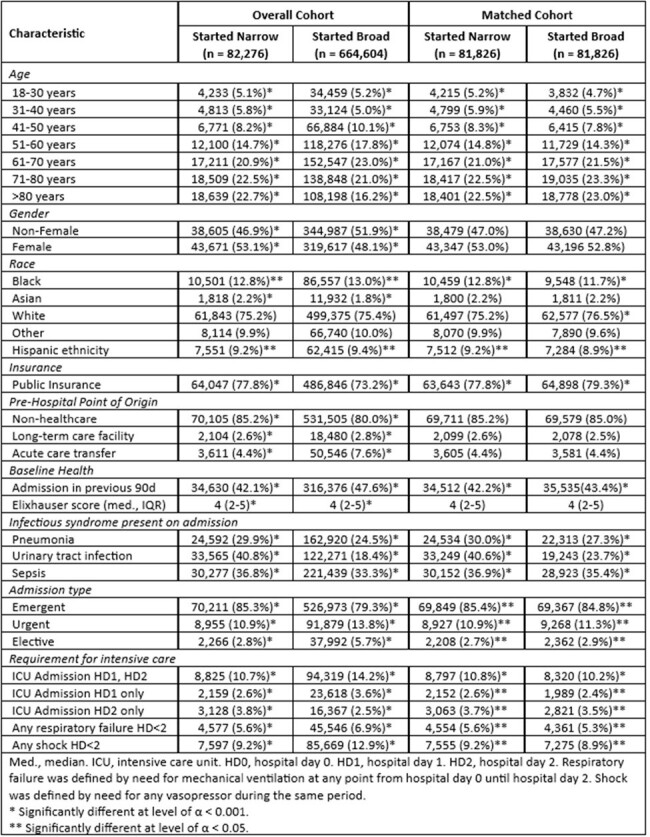
Characteristics of Patients in the Overall and Matched Cohorts

**Conclusion:**

In a large propensity score-matched analysis of observational clinical data, starting empiric narrow-spectrum antibiotic therapy and needing to escalate to broad-spectrum antibiotic therapy later was not associated with worse clinical outcomes. Our findings challenge traditional guidance on how to select empiric antibiotic coverage.

**Table 2.**
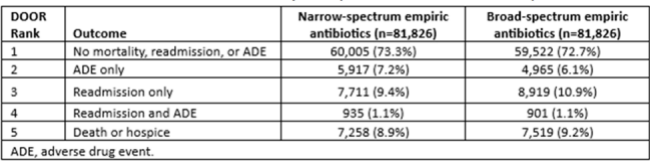
Distribution of DOOR by Empiric Treatment Group in the Matched Cohort

**Disclosures:**

**Kimberly C. Claeys, PharmD, PhD**, bioMérieux: Advisor/Consultant|bioMérieux: Honoraria **Anthony Harris, MD, MPH**, Innoviva: Advisor/Consultant|UpToDate: Infection Control Editor

